# The impact of stator module gaps on the performance of canned permanent magnet synchronous motors with segmented stators

**DOI:** 10.1038/s41598-025-13864-w

**Published:** 2025-08-19

**Authors:** Li Ming, Zheng Jixin, Du Guanghui, Lun Shuxian

**Affiliations:** 1 School of Control Science and Engineering, Bohai University, Jinzhou, 121013 China; 2 School of Electrical and Control Engineering, Xi’an University of Science and Technology, Xi’an, 710054 China

**Keywords:** Energy science and technology, Engineering, Mathematics and computing

## Abstract

The segmented stator structure is applied to the canned permanent magnet synchronous motor (CPMSM) can improve the performance of the motor. However, the specific and detailed effects of varying stator module gaps on CPMSM performance, particularly efficiency and torque, require further investigation. In this paper, an iterative calculation method for the electromagnetic performance of the motor is proposed. Using this method, the influence of stator module gap on the electromagnetic performance of the CPMSM with segmented stators is analyzed under the conditions of constant current and constant torque. The calculation results are compared with the finite element calculation results. The results show that under constant current, the increase of the stator module gap leads to the decrease of the loss, torque, efficiency and power factor. Under the condition of constant torque, with the increase of the stator module gap, the loss and power factor of the CPMSM are reduced, but the efficiency of the CPMSM is gradually improving. Finally, a prototype is made and a test platform is built. The test results verify the correctness and effectiveness of the proposed method and results. This study not only provides new insights into the complex interactions between module gap size, flux distribution, harmonic content, loss mechanisms, torque output, and efficiency in this specific motor structure, but also offers guidance for the optimized design of the CPMSM.

## Introduction

In the modern industrial landscape, vacuum pumps are indispensable in a variety of critical sectors, including petroleum, chemical engineering, semiconductor manufacturing, and the nuclear industry. According to recent statistical data, the global market for vacuum pumps is witnessing consistent growth, with projections estimating a market value in the tens of billions of dollars by 2027^1^. This growth underscores not only the significance of vacuum pumps across various industries but also the pressing demand for enhancing equipment efficiency.

At the heart of vacuum pumps lies the canned permanent magnet synchronous motor (CPMSM)^2–4^, which serves as the primary driver. The CPMSM efficiency is pivotal as it directly impacts the performance and energy consumption of the vacuum pump system. However, the presence of a can sleeve in the CPMSM can lead to eddy current losses^5^, which reduce the CPMSM efficiency compared to the traditional permanent magnet synchronous motor (PMSM)^6^.

To improve the efficiency of the CPMSM, researchers have introduced the CPMSM with segmented stators^7^. The stator of this motor is designed with a modular structure, which allows for a higher slot filling factor. Extensive research has been conducted by researchers on the stator modular PMSM^8–11^, including the motor design, performance analysis, the impact of manufacturing tolerances, and computational models for electromagnetic performance.

References^8,9^ studied the impact of manufacturing tolerances on the performance of PMSMs, particularly the effect of stator segmentation on output torque and torque ripple. These studies evaluated the specific impact of different manufacturing tolerances on motor performance through finite element analysis (FEA) and design of experiments (DOE), and proposed methods to reduce the number of design parameters to simplify the sensitivity analysis process. An analytical model is proposed for the rapid and accurate calculation of the electromagnetic performance of segmented PMSMs with large angular gaps^10^. These models are applicable to PMSMs with wide air gaps, large diameters, multiple pole numbers, and large angular gaps, and their accuracy is verified by comparing the results with two-dimensional finite element method (FEM). Reference^11^ explored the impact of stamping during the manufacturing process on the inherent torque ripple of segmented PMSMs. The study found that material degradation caused by stamping can introduce additional low-order harmonics in the motor teeth, which may be comparable to, or even greater than, the inherent torque ripple.

According to the current literature, while research on segmented stator permanent magnet synchronous motors (PMSM) has achieved some advancements, research concerning the effects of stator module gaps on the electromagnetic performance of the CPMSM is comparatively limited. Given the distinctive structure of the CPMSM, it is not yet clear whether the patterns of influence of stator module gaps on the electromagnetic performance of the PMSM are applicable to the CPMSM, necessitating more thorough analysis and quantitative studies. Furthermore, current studies are predominantly concentrated on theoretical analysis and simulation, whereas research that compares these theoretical outcomes with experimental data is even more scarce. Thus, this research aims to bridge this gap by integrating iterative calculation method (ICM) with experimental verification, to address this research void and offer novel theoretical backing and practical direction for the design and performance optimization of the CPMSM.

The structure of this paper is as follows. In the second section, the calculation method of electromagnetic performance is introduced. Then, in the third section, the influence of the stator module gap on the electromagnetic performance is studied under the conditions of constant current and constant torque. Finally, the fourth section compares the calculation results with the prototype test results, and verifies the correctness of the conclusion.

## Electromagnetic performance calculation method

**Fig. 1:**
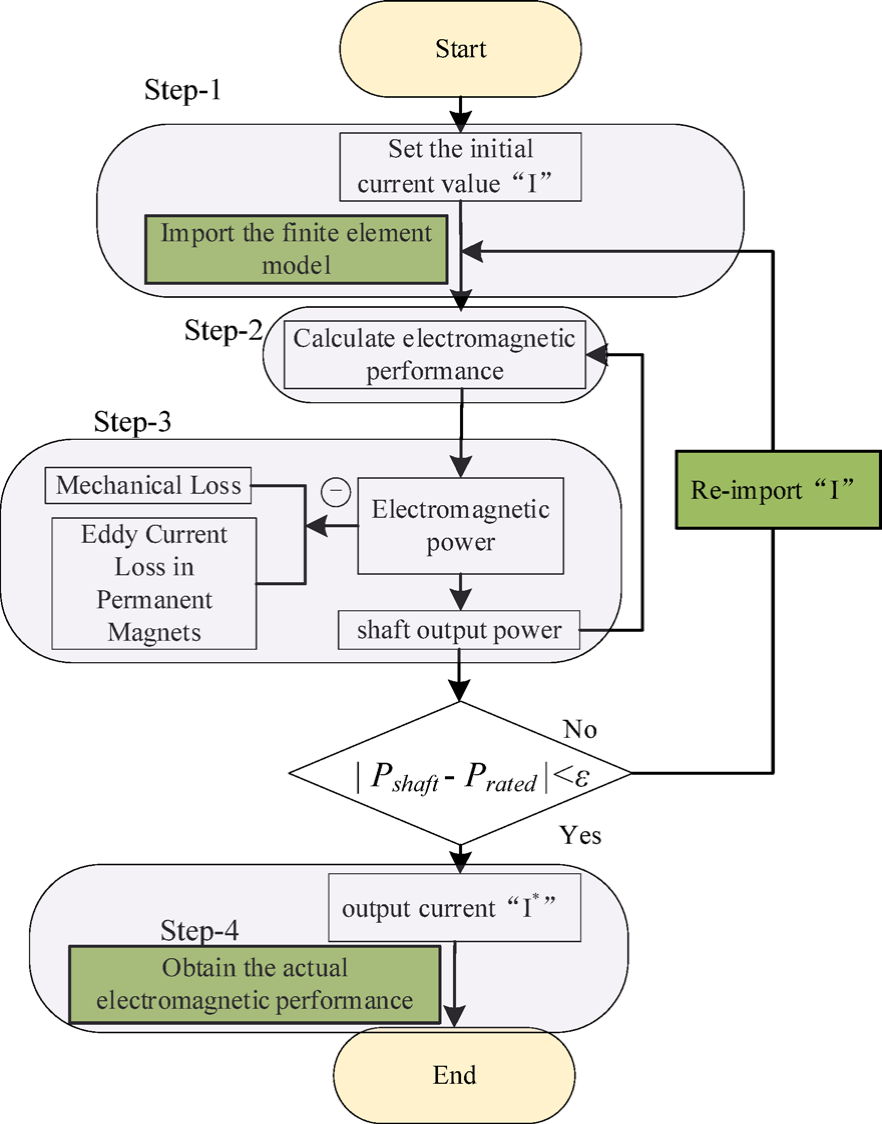
Flowchart of the ICM.

The FEM is widely recognized for its ability to simulate the electromagnetic power of motors; however, it falls short in accurately computing mechanical losses, stray losses, and friction losses, which constitute a significant portion of the total motor losses^12^. Consequently, the torque derived from simulations does not reflect the actual output torque of the motor shaft. To overcome the limitation of the FEM, an iterative calculation method (ICM) is proposed in this paper. The ICM builds upon standard finite element analysis by incorporating an iterative loop to account for mechanical losses and eddy current losses in permanent magnets. ICM adjusts the simulation current iteratively to ensure that the simulation results match the power balance state of actual motor operation, bringing the simulation results closer to the actual running conditions of the motor. This breakthrough overcomes the limitations of traditional FEM in simulating the motor performance. Figure 1. illustrates the computational flowchart of the ICM.

As shown in Fig. 1, the first step is to use the rated current of the CPMSM as the initial excitation of the finite element model. The second step is to determine the electromagnetic power of the CPMSM using finite element model. In the third step, according to the motor speed, the mechanical loss of the motor is estimated by empirical formula, and the eddy current loss in the permanent magnets is calculated by finite element model. This two losses are then subtracted from the electromagnetic power to determine the actual output power on the motor shaft. The output power on the shaft is compared with the rated power. When the error between the two is larger than the specified threshold, the simulation current is increased by 1% and recalculated. Choosing 1% as the step size for current adjustment is a decision made after considering both computational accuracy and convergence speed. If the step size is too large, it may lead to non-convergence or slow convergence in the iterative process. If the step size is too small, it will increase the number of iterations and the computational time required. When the error between the two is less than the specified threshold (ε = 0.1%), the corrected simulation current I^*^ is output. Additionally, to prevent the iterative process from failing to converge, a maximum number of iterations has been set to 10. The fourth step is to obtain the electromagnetic performance of the CPMSM using I^*^, when the iteration converges.

To verify the effectiveness of the proposed ICM, this paper takes a 1.5 kW CPMSM with segmented stators as the research object. The canned sleeve material is austenitic stainless steel 304 with high corrosion resistance and high mechanical strength. The selection of can sleeve material has an important influence on the performance of the canned motor. Using ferritic stainless steel can limit the can loss, but its ferromagnetism will lead to the increase of magnetic leakage of motor and affect the utilization rate of permanent magnets. Therefore, austenitic stainless steel 304 is selected as the material of the motor can sleeve. The thickness of the canned sleeve and the rotor sleeve are both 0.5 mm, and the grade of the permanent magnet is N38UH. The main parameters of the CPMSM are shown in Table 1.

**Table 1:** Basic parameters of the modular CPMSM.

Parameters	Value
Rated power(kW)	1.5
Phase number	3
Pole number	6
Rated frequency (Hz)	450
Can thickness (mm)	0.5
Stator slots	9
Stator length(mm)	23
Stator outside diameter(mm)	107
Rotor outside diameter(mm)	46.5

As can be seen from Table 1, the rated operating frequency of the motor is 450 Hz, and the inverter loss caused by high frequency should be paid attention to. However, the purpose of this study is to analyze the influence of stator module gaps on the CPMSM performance. In order to simplify the calculation, the inverter loss is not considered in this study.

**Fig. 2:**
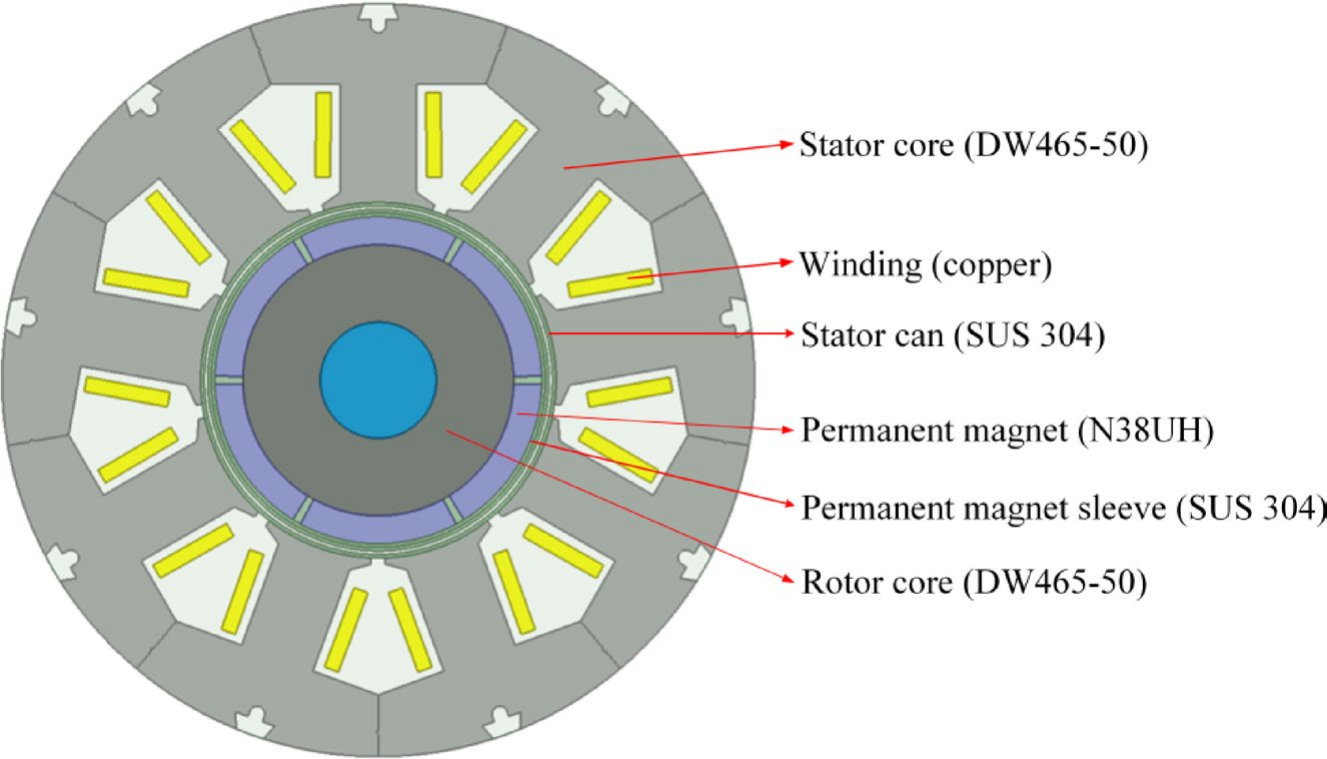
The Finite element model of the 1.5 kW CPMSM.

In this study, ANSYS EM Suite 2023R1 was used as the finite element software. Based on the parameters of the motor in Table 1, the 2D finite element model of the 1.5 kW CPMSM is shown in Fig. 2. Due to the combined effects of material properties, machining processes, and assembly accuracy in the actual manufacturing process, the non-uniformity of the stator module gaps is inevitable. However, accurately simulating these non-uniform air gaps is very difficult during modeling and is computationally expensive. Therefore, we assumed a uniform air gap in the finite element model to simplify the analysis and maintain computational efficiency. Although this assumption hardly reflects the complexity in actual manufacturing, it can reasonably approximate the overall performance trend of the actual motor, providing valuable references for design and optimization. Therefore, in Fig. 2, the finite element model adopts a uniform air gap, and the stator module is modeled parametrically, allowing the air gap between stator modules to be adjusted by changing the parameter variables. In addition, to simplify the analysis of the CPMSM, the following assumptions were made in this paper^13^:

1. The displacement current and skin effect in stator winding are ignored;
2. The influence of temperature on the permeability and conductivity of materials is ignored.

The governing equation and boundary condition of the analysis model are as follows^14,15^:

1 $$\left\{ {\begin{array}{*{20}{c}} {\frac{\partial }{{\partial x}}\left( {\nu \frac{{\partial {A_Z}}}{{\partial x}}} \right)+\frac{\partial }{{\partial y}}\left( {\nu \frac{{\partial {A_Z}}}{{\partial y}}} \right)= - {J_s}+\sigma \frac{{d{A_Z}}}{{dt}}} \\ {{{\left. {{A_Z}} \right|}_\Gamma }=0} \end{array}} \right.$$

where Az is magnetic vector potential, ν is permeability, σ is conductivity, *J*_s_ is current density, Γ is outer boundary.

## The impact of stator module clearance on the performance of canned permanent magnet shielding motors

With the improvement of motor manufacturing technology, the gap between the motor stator modules is improved. Therefore, this paper assumes that the maximum gap between the stator modules is 0.5 mm. The proposed ICM in this paper and FEM are used to analyze the influence of the stator modular gap on the electromagnetic performance of the CPMSM with segmented stators under the two conditions of the constant input current and the constant output torque.

**Fig. 3:**
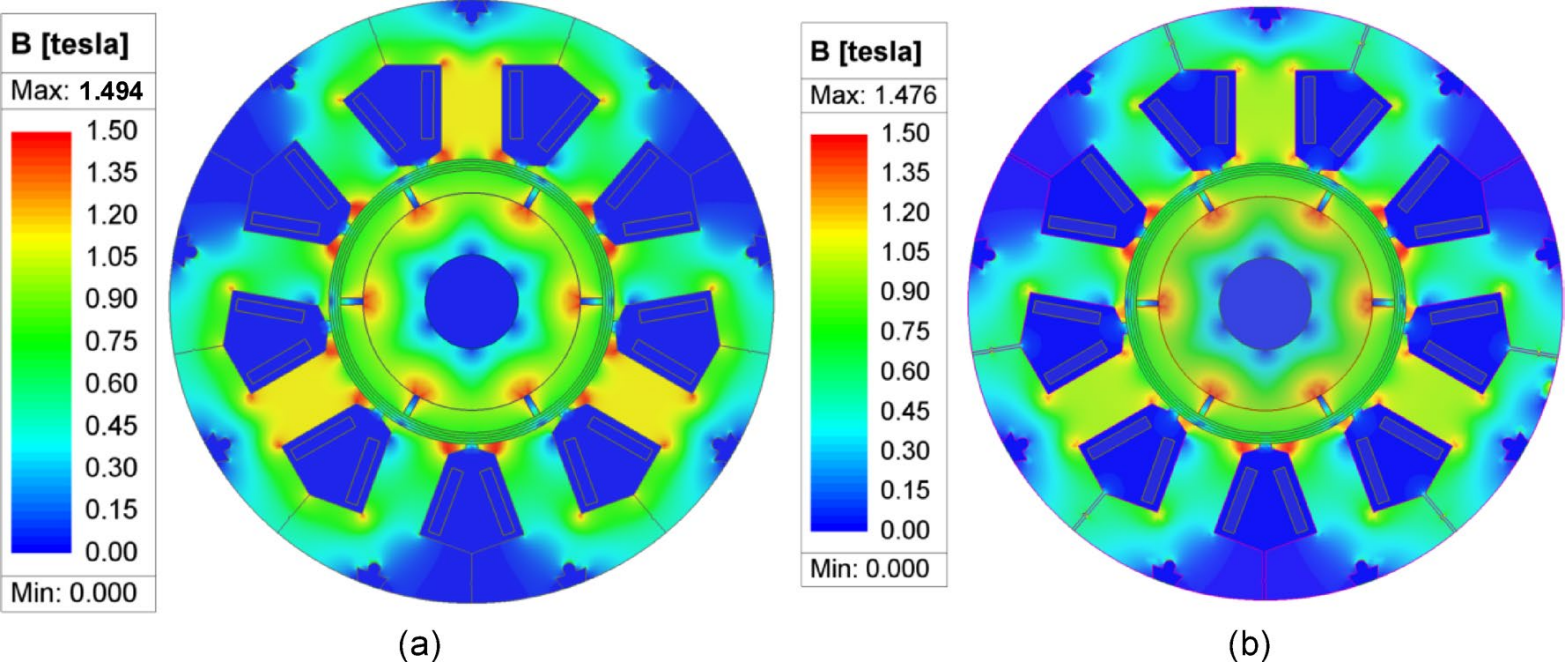
Flux density distribution of the CPMSM under no-load condition. (**a**) the gap is 0 mm; (**b**) the gap is 0.5 mm.

Firstly, the distribution of magnetic field is analyzed when there is no gap between the stator modules and the gap is 0.5 mm during the no-load operation of the motor. The results are shown in Fig. 3.

As depicted in Fig. 3, the flux density distribution within the CPMSM is relatively uniform, and its amplitude is low, which is designed to mitigate the can loss. Furthermore, Fig. 3 clearly demonstrates that an increase in the stator module gap leads to a significant reduction in the magnetic flux density within the motor. This indicates that the stator module gap has a distinct modulating effect on the flux density, and effectively alters the magnetic field inside the motor by adjusting the magnetic reluctance of the magnetic path.

**Fig. 4:**
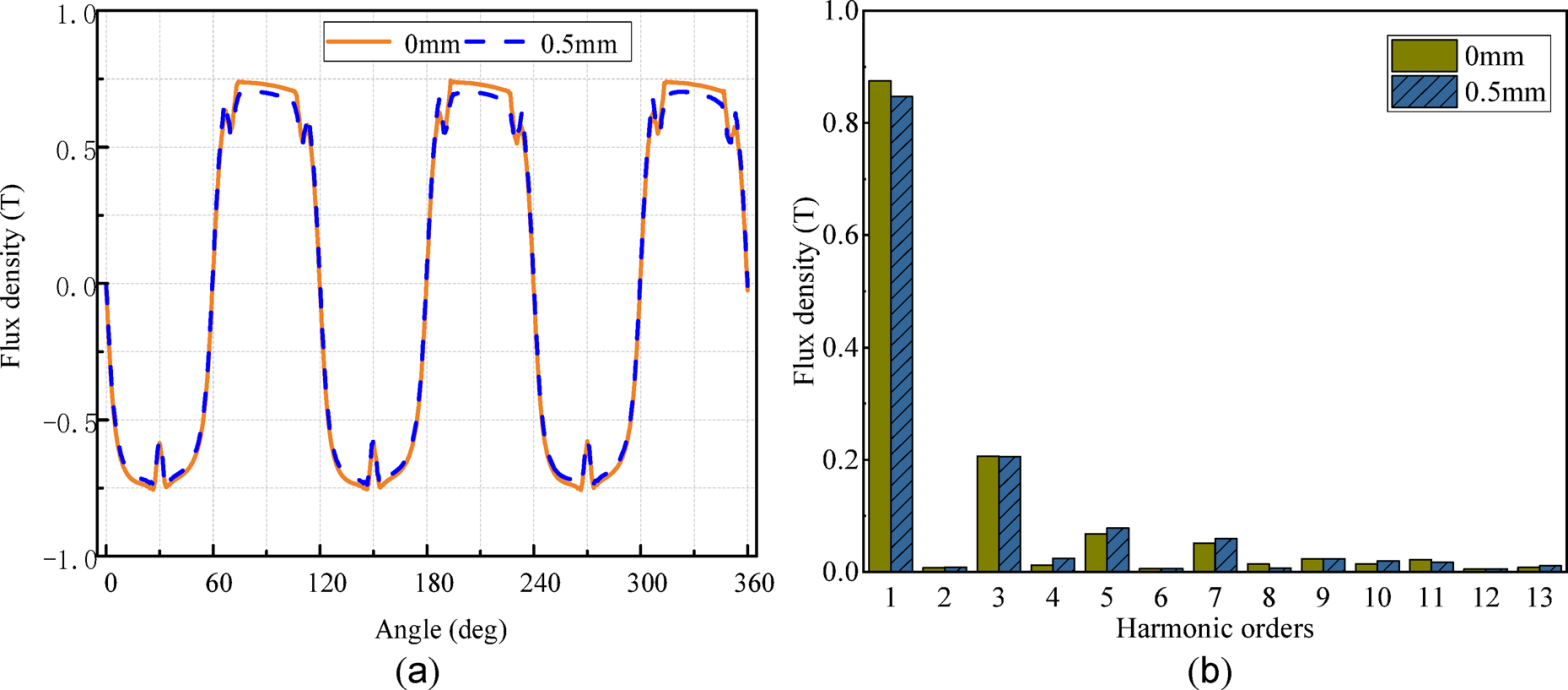
The air gap flux density of the CPMSM. (**a**) the waveform; (**b**) the harmonic components.

To further analyze the influence of the stator module gap on the electromagnetic field of the CPMSM^16^, the influence of the stator module gap on the air gap flux density of the motor in no-load operation of the CPMSM is calculated. The result is shown in Fig. 4.

Figure 4 illustrates that an increase in the stator module gap results in a reduction of the fundamental amplitude of the air-gap flux density. Specifically, with a zero gap, the fundamental amplitude is 0.875 T, which decreases to 0.847 T when the gap is increased to 0.5 mm, representing a 3.2% reduction. Notably, while the fundamental is diminished, the stator module gap simultaneously leads to an increase in the 5th, 7th, and 13th harmonic of the air-gap flux density. This alteration in the flux density harmonic profile consequently influences the loss distribution, electromagnetic torque, power factor, and overall efficiency of the CPMSM.

## Research under the condition of constant input current

To ensure that the motor operates within a safe temperature range, it is necessary to maintain a constant input current to the motor. Therefore, with the input current constant, the proposed ICM method and FEM were used to analyze the impact of the stator module gap on the electromagnetic performance of the CPMSM^17^.

1. The impact of stator module gaps on the can loss

**Fig. 5:**
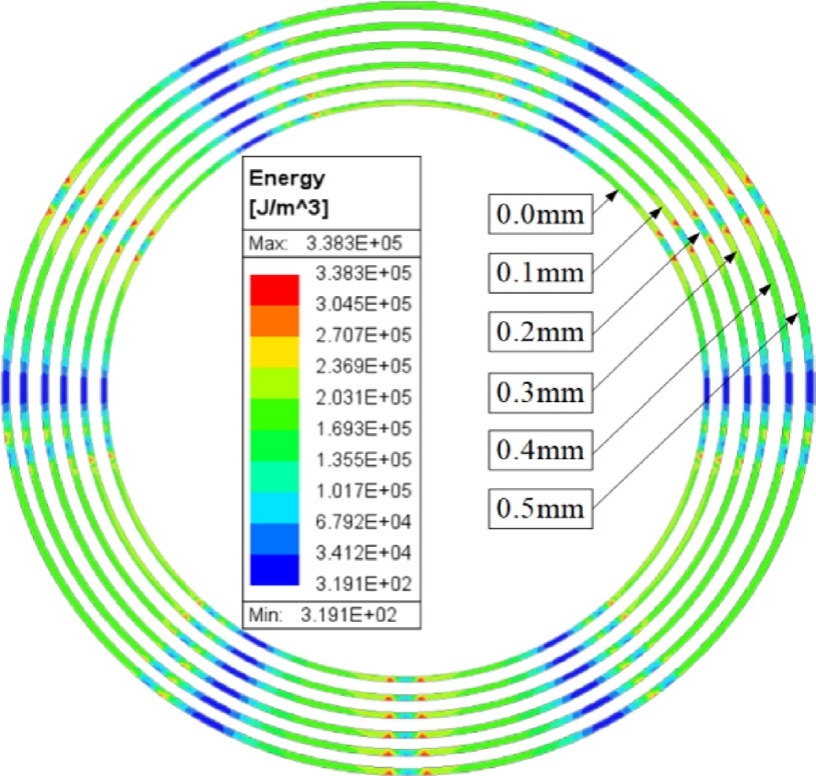
The distribution of eddy current loss density of the can sleeve.

Considering that the can loss accounts for 30%-40% of the total loss of the CPMSM^18^, this paper analyzes the eddy current loss density of can sleeve under different stator module gaps. Figure 5 shows the distribution of eddy current loss density of the can sleeve for stator module gaps varying from 0.0 mm to 0.5 mm. According to Fig. 5, it can be seen that the distribution law of eddy current density of the can sleeve under different stator module gaps is basically the same.

**Fig. 6:**
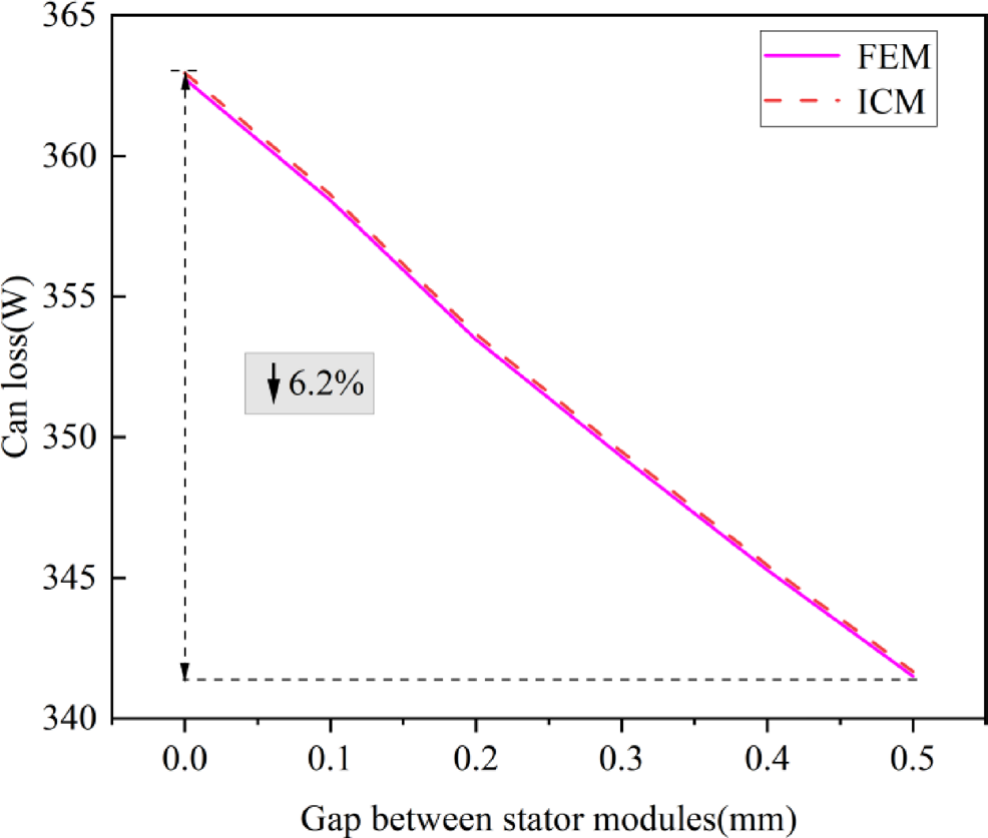
Influence of stator module gap on can loss.

Figure 6 shows the can loss calculated by FEM and ICM under different stator module gaps.

In Fig. 6 that the maximum difference between the can loss calculated by the FEM and the ICM is 0.5%. This shows that after considering the mechanical loss and eddy current losses in permanent magnets, the can loss of the CPMSM changes little.

In addition, Fig. 6 shows that under the condition of constant input current, the can loss decreases with the increase of the stator modular gap. Taking the proposed ICM in this paper as an example, the can loss is 364.18 W when the stator gap is 0 mm. When the gap increases to 0.5 mm, the can loss decreases to 341.62 W, a decrease of 6.2%. The can loss is reduced because the increase in the stator module gap results in a decrease in magnetic flux density.

2) The impact of stator module gaps on the stator core loss

**Fig. 7:**
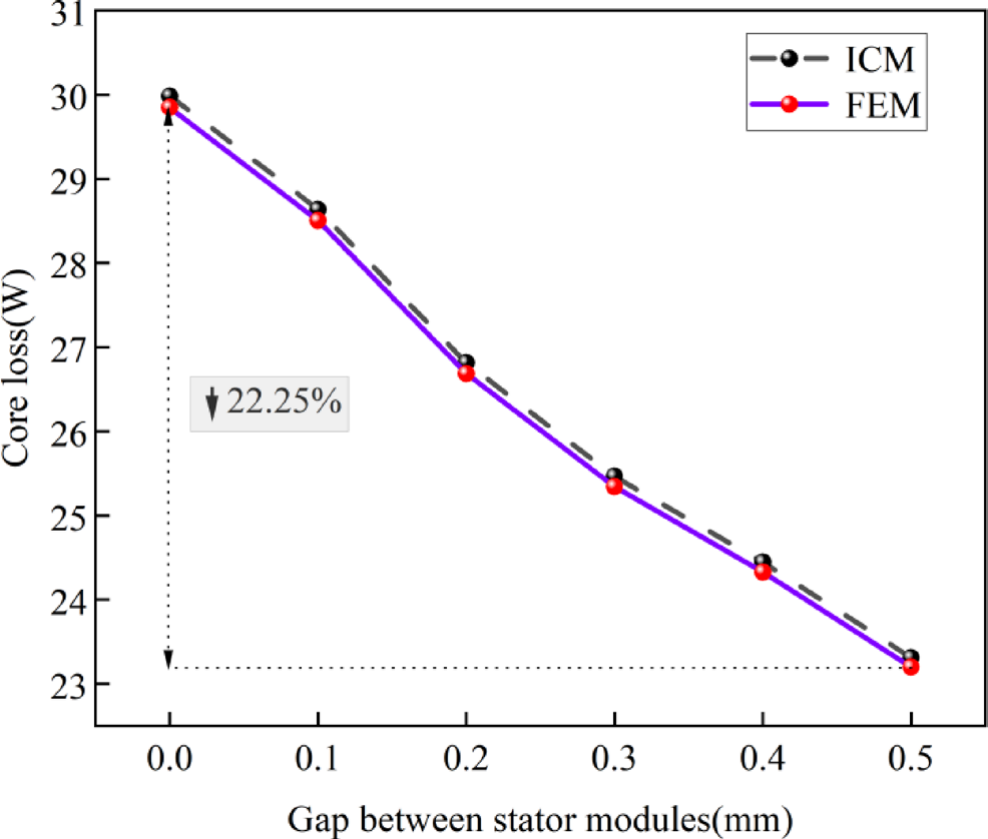
Effect of stator module gap on the core loss.

Then, the effects of stator module gaps on the core loss was examined through the ICM and the FEM, as illustrated in Fig. 7.

From Fig. 7, it can be seen that the maximum difference between the core loss calculated by the proposed ICM and the FEM is 5.8%, which indicates that mechanical losses and eddy current losses in permanent magnets have a certain impact on the core loss of the CPMSM.

It can also be seen from Fig. 7 that with the input current constant, the core loss gradually decreases as the stator module gap increases. Taking the calculation results of the CIM proposed in this paper as an example, when the stator module gap is 0 mm, the core loss is 30.01 W. When the stator module gap is increased to 0.5 mm, the core loss is 23.31 W, a reduction of 22.25%. This is related to the increase in stator module gap and the decrease in flux density. Furthermore, Fig. 7 also shows that the variation of the core loss at different gap values is not a simple linear relationship, and this characteristic should be taken into account in the motor design.

3) The impact of stator module gaps on the output torque of the CPMSM

**Fig. 8:**
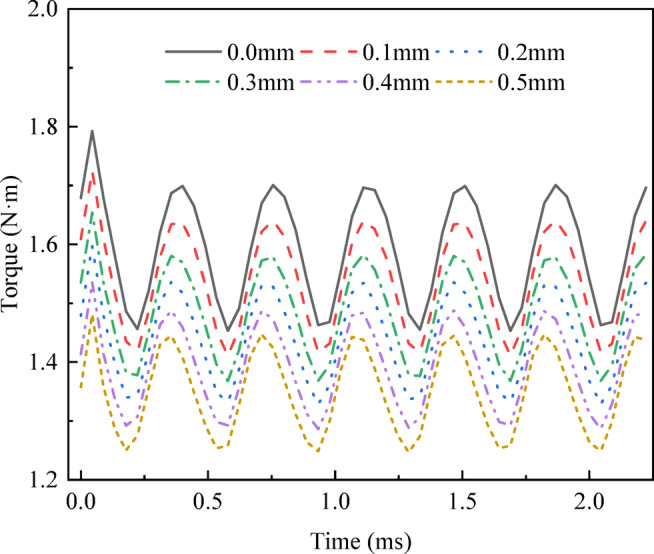
Effect of stator module gap on the CPMSM torque.

The influence of the stator module gap on the motor torque is analyzed by using the proposed ICM in this paper^19^. The results are shown in Fig. 8.

The results of Fig. 8 show that when the input current is constant, with the increase of the stator module gap, the average torque of the CPMSM decreases gradually, and the torque ripple also decreases gradually. When the gap between stator modules is 0 mm, the torque of the CPMSM is 1.59 N·m, and the torque ripple is 14.69%. When the gap between stator modules is 0.5 mm, the torque is reduced to 1.35 N·m, a decrease of 15.1%. The torque ripple is reduced to 11.89%, a decrease of 2.8%. The decrease of torque and torque ripple is directly related to the increase of stator module gap, and the decrease of fundamental flux density and harmonic flux density respectively. Although increasing the stator module gaps can reduce torque ripple, it can also lead to a decrease in output torque of the CPMSM. Therefore, in practical applications, increasing the stator module gap is a design decision that requires a trade-off between reducing the motor torque ripple and performance degradation.

4) The influence of stator module gap on the efficiency and power factor of the CPMSM

Efficiency and power factor are the key indexes of reliable and economical operation of the motor, so the influence of stator module gap on the CPMSM efficiency and power factor is analyzed.The efficiency of the CPMSM is calculated by^20^.

2 $$\eta =\frac{{T \cdot \Omega }}{{T \cdot \Omega +{P_{cu}}+{P_{fe}}+{P_{can}}+{P_{add}}}}$$

where *T* is torque, Ω is mechanical angular velocity, *P*_*cu*_ is the copper loss refering to Eq. (3), *P*_*fe*_ is the core loss, *P*_*can*_ is the can loss, and *P*_*add*_ is the additional loss.

As the calculation of copper loss in this study can only be obtained by finite element method, the current finite element software can not accurately calculate the increase of copper loss caused by skin effect caused by high frequency, so this paper does not consider the influence of frequency on copper loss of the motor.

3 $$P_{{cu}} = mI_{{ph}}^{*2} R_{{ph}}$$

where *m* is the phase number of the CPMSM, I^*^_ph_ is the input phase current after iterative calculation, *R*_*ph*_ is the phase resistance.

The term “additional losses " refers to the extra loss generated under non-ideal conditions, including manufacturing defects, uneven load and harmonic effects. The accurate calculation of the additional loss is complicated. For the convenience of calculation, it is usually assumed that the additional loss is about 0.5% of the motor output power^21^.

The power factor of a motor can be determined by the phase angle between voltage and current, provided that both the voltage and current waveforms are sinusoidal. The load current of a motor is typically non-sinusoidal, and using this method could introduce errors. Therefore, the calculation of the power factor is calculated by Eq. (4)^22^.

4 $$\cos \varphi =\frac{{T \cdot \Omega +{P_{cu}}+{P_{fe}}+{P_{can}}+{P_{add}}+{P_\vartriangle }}}{{\sqrt 3 {U_N}{I_N}}}$$

where *U*_*N*_ is the rated voltage, *I*_*N*_ is the rated current, *P*_△_ is the total of mechanical and friction losses.

**Fig. 9:**
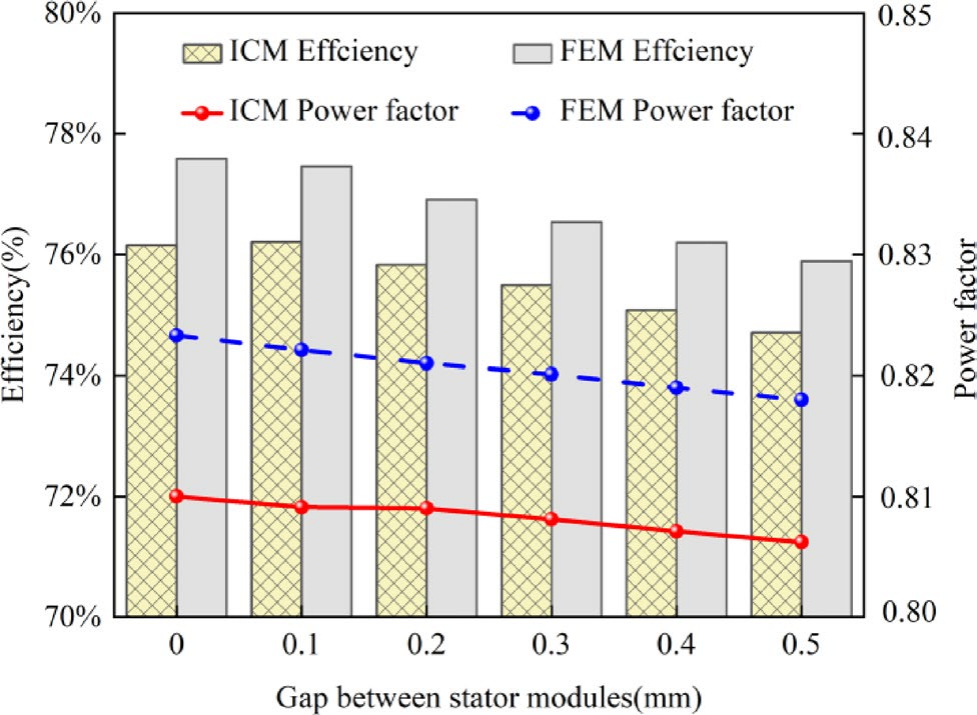
The impact of air gap between stator modules on motor efficiency and power factor.

Figure 9 shows the relationship between stator module gap and the CPMSM efficiency and power factor under the condition of constant current.

As can be seen from Fig. 9, the efficiency and power factor calculated by the ICM are lower than those calculated by the FEM, because the ICM considers the mechanical loss and permanent magnet loss, which is closer to the actual operation of the motor. Additionally, Fig. 9 also shows that as the stator module gap increases, the efficiency and power factor of the CPMSM gradually decrease. Taking the calculation results of the ICM as an example, when the stator module gap is 0 mm, the efficiency of the CPMSM is 76.16%, and the power factor is 0.81. When the stator module gap is increased to 0.5 mm, the efficiency of the CPMSM drops to 74.87%, a decrease of 1.29%. However, the power factor has hardly changed. The reasons for the change in power factor are complex. The increase in the stator module gap indirectly affects the power factor by influencing multiple factors such as the magnetic flux path, inductance, core losses, and copper losses.

According to the above results, although the increase of stator module gap reduces the core loss and the can loss, the output torque of the CPMSM is also decreasing, and the reduction value of the loss is less than that of the output power, which leads to the decrease of the CPMSM efficiency. In addition, the stator module gap has little influence on the power factor.

## Research on CPMSM under the condition of the constant output torque

To ensure the safety and reliability of the vacuum pump system, the CPMSM is required to have a constant output torque. It should be noted that achieving the same electromagnetic torque requires either maintaining the same average flux density or increasing the current. To achieve the same average flux density, it would require increasing the quantity of permanent magnets or upgrading their grade, which is not allowed for motors that have already been manufactured or in cases where cost is a constraint. Therefore, this study achieves the same electromagnetic torque by increasing the current. Using the ICM, the relationship between the stator module gap and the input current is shown in Table 2 under the condition of the same output torque of the CPMSM.

**Table 2:** Current values for the same Torque.

Stator Module gap(mm)	0.0	0.1	0.2	0.3	0.4	0.5
current (A)	8.4	8.6	8.8	9.0	9.2	9.5

Table 2 shows that the motor current gradually increases with the increase of the stator module gap. The increase of current will inevitably lead to the change of the motor loss and performance. Therefore, under the condition of the same motor output torque, the influences of the stator module gap on copper loss, can loss, core loss, efficiency and power factor of the CPMSM are analyzed.

1. The influence of the stator module gap on the copper loss

**Fig. 10:**
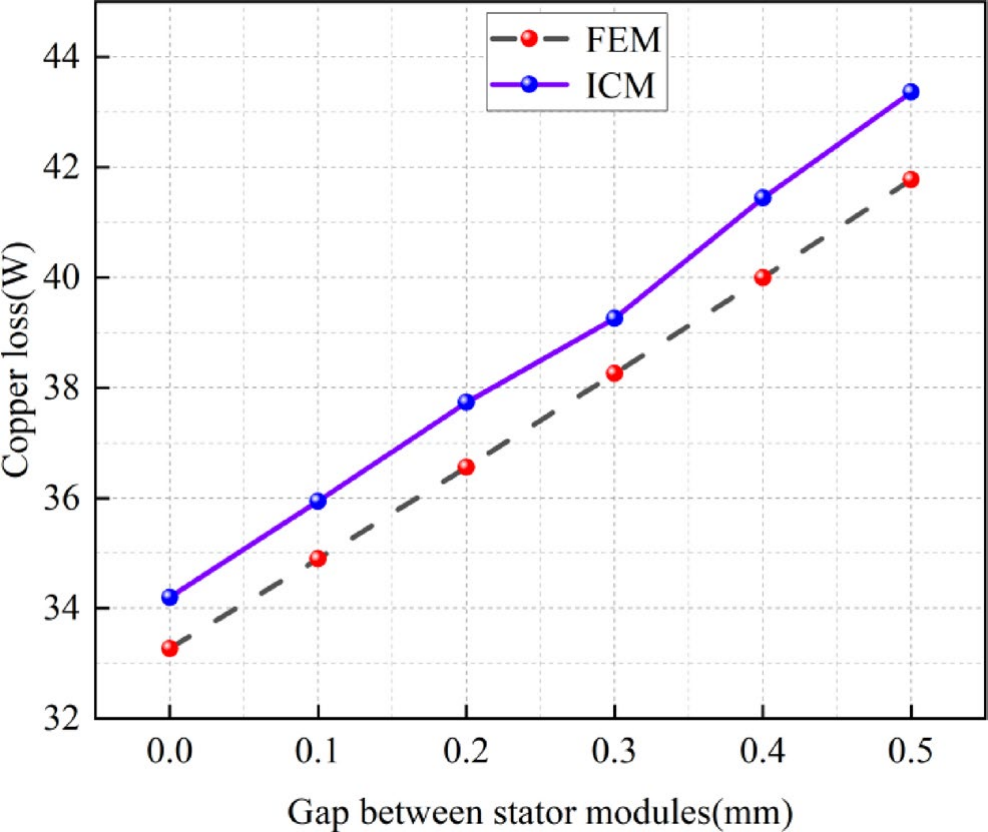
The influence of stator module gaps on copper losses.

Figure 10 shows the influence of the stator module gap on the copper loss of the CPMSM by the FEM and the ICM. From Fig. 10, it can be observed that the copper loss obtained through the ICM is slightly greater than that obtained through the FEM. This is because the ICM takes into account the mechanical losses and magnet losses and corrects the simulation current accordingly, with the maximum difference between the two being 3.59%.

Additionally, Fig. 10 indicates that as the stator module gap gradually increases, the copper loss of the CPMSM also gradually increases. From Table 2, it can be seen that this is due to the increase in stator current. Taking the ICM calculation results as an example, when the stator gap is 0 mm, the copper loss is 34.2 W, and when the stator gap is 0.5 mm, the copper loss is 43.4 W. As the stator gap increases from 0 to 0.5 mm, the copper loss increases by 6.8 W, which is a 20% increase.

2) The influence of stator module gap on the can loss

**Fig. 11:**
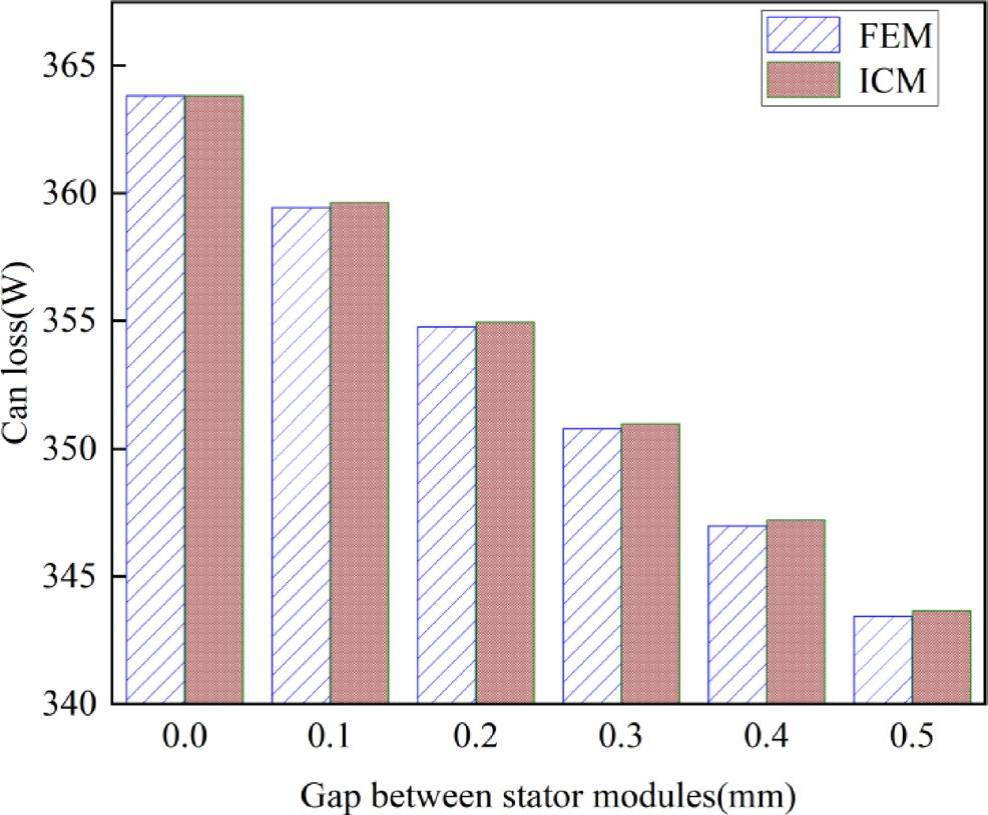
Influence of the stator module gap on the can loss.

Then, under the condition of constant motor output torque, the influence of stator module gap on can loss is analyzed by the FEM and the proposed ICM. The results are shown in Fig. 11.

Figure 11 shows that the can loss obtained using the FEM and the ICM is not significantly different, with the maximum difference between the two being only 0.2%.

Additionally, Fig. 11 also indicates that with the output torque constant, the can loss gradually decreases as the stator module gap increases. Taking the ICM results as an example, when the stator module gap is 0 mm, the can loss is 364.18 W, and when the stator gap is 0.5 mm, the can loss is 343.43 W. When the stator module gap increases from 0 mm to 0.5 mm, the can loss decreases by 20.75 W, which is a reduction of 5.7%.

3) The influence of the stator module gap on the core loss

**Fig. 12:**
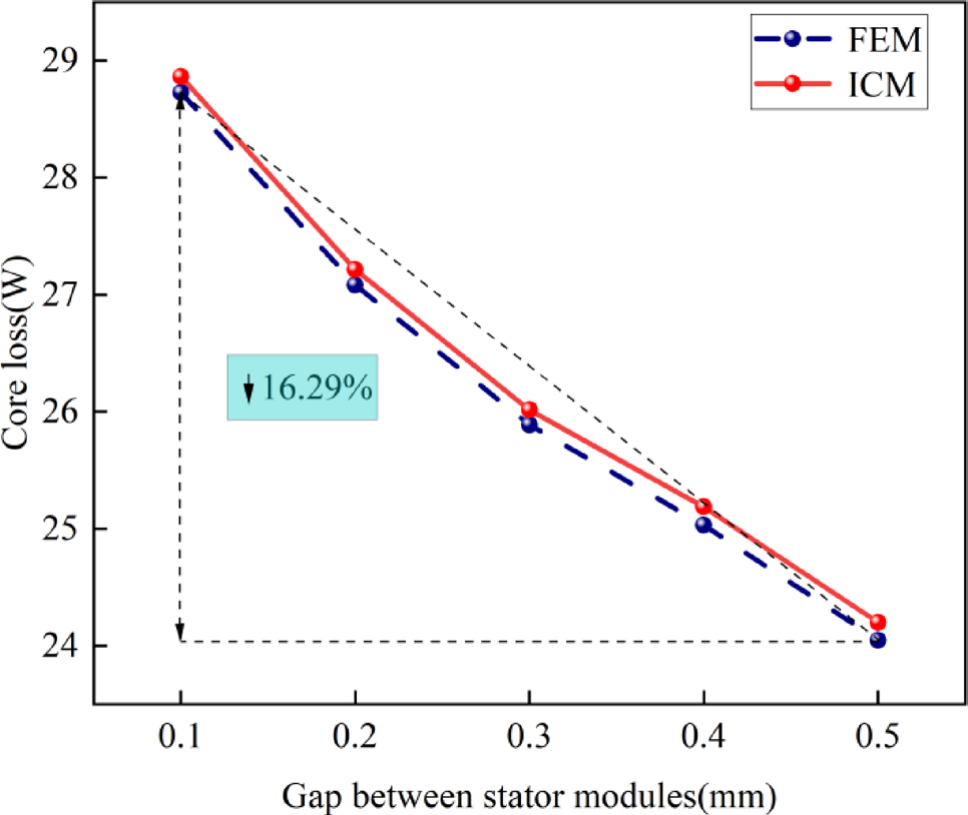
The relationship between the stator module gap and the core loss.

Figure 12 shows the relationship between the stator module gap and the core loss under the constant output torque of the CPMSM.

Figure 12 shows that the core loss obtained by the FEM and the proposed ICM is still not very different, and the maximum difference between them is 0.1%.

In addition, Fig. 12 shows that as the stator module gap gradually increases, the core loss gradually decreases. Taking the ICM results as an example, when the stator module gap is 0.1 mm, the core loss is 28.7 W, and when the stator module gap is increased to 0.5 mm, the core loss is 24 W. As the stator gap increases from 0.1 mm to 0.5 mm, the core loss is reduced by 4.7 W, which is a decrease of 16.3%.

4) The influence of the stator module gap on efficiency and power factor of the CPMSM

**Fig. 13:**
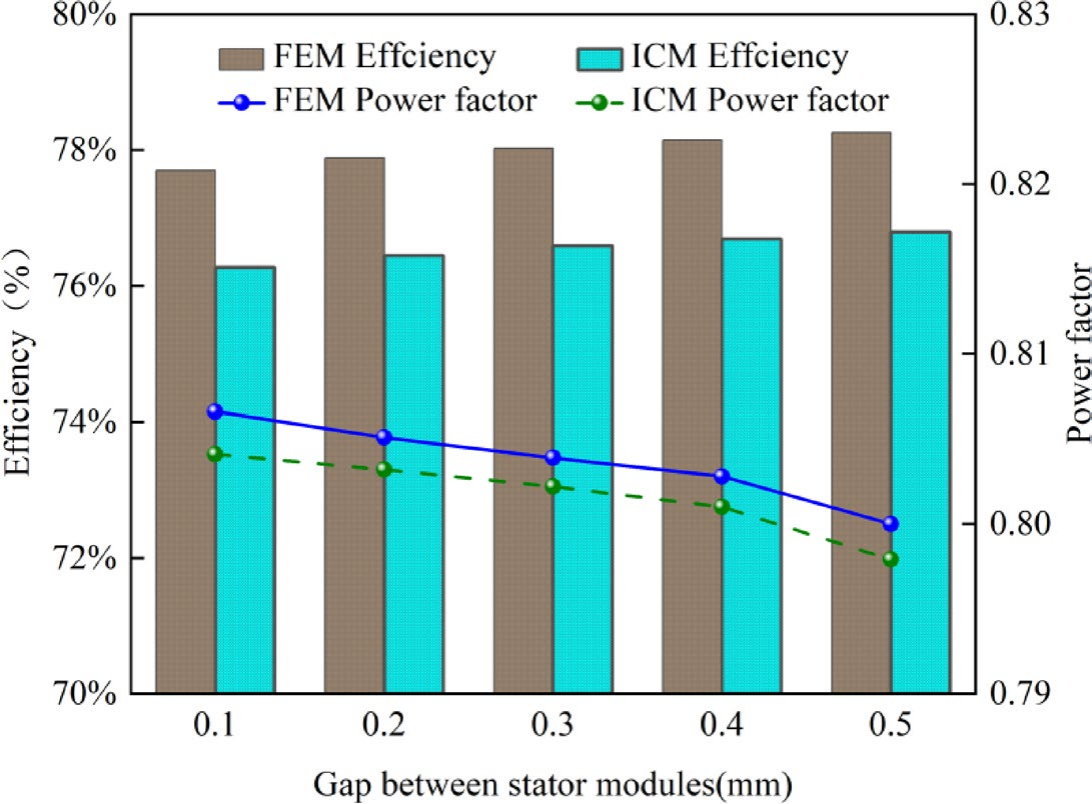
The impact of air gap between stator modules on motor efficiency and power factor.

Based on the aforementioned analysis, under the condition of constant output torque of the CPMSM, the impact of the stator module gap on the efficiency and power factor of the CPMSM was analyzed using the ICM and the FEM, with the results shown in Fig. 13.

From Fig. 13, it can be seen that under the condition of constant output torque, the efficiency and power factor obtained using the ICM proposed in this paper are lower than those obtained using the FEM. Furthermore, as the stator module gap increases, the efficiency gradually increases, while the power factor gradually decreases. Taking the ICM results as an example, when the stator module gap is 0 mm, the motor efficiency is 76.16%, and the power factor is 0.81. When the stator module gap is increased to 0.5 mm, the motor efficiency is improved to 76.78%, an increase of 0.62%, and the power factor decreases to 0.80, a decrease of 1.49%.

The efficiency increases because with the increase of the stator module gap, the core loss and the can loss decrease, and the output power is constant. The decrease of power factor is caused by the increase of magnetic leakage caused by the increase of stator module gap.

## Experimental verification

**Fig. 14:**
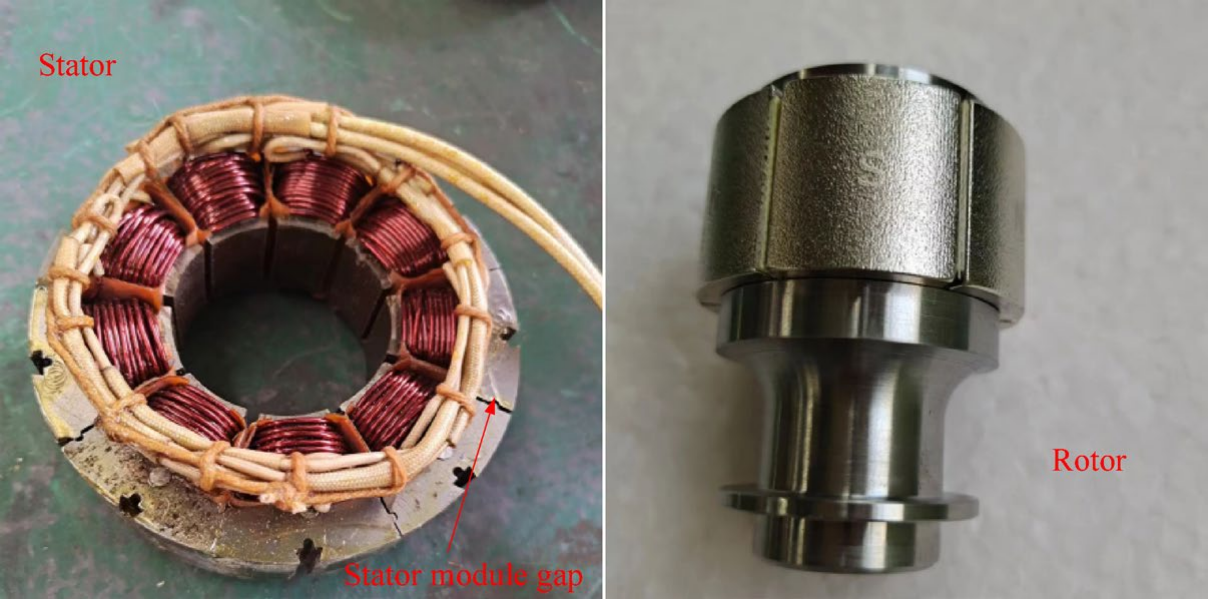
The CPMSM prototype.

To verify the accuracy of the proposed ICM and results, a prototype was manufactured based on the parameters in Table 3, as shown in Fig. 14. The rated power of the motor is 1.5 kW, the rated current is 8.4 A, and the rated voltage is 200 V. It is worth noting that due to the combined effects of material properties, machining processes, and assembly accuracy in the actual manufacturing process, the non-uniformity of the stator module gaps in the prototype is inevitable. However, to simplify the finite element analysis and maintain computational efficiency, we assumed a uniform air gap in the simulation model. Although this assumption does not fully reflect the complexity of the actual prototype, it is reasonable in approximating the overall performance trend and can provide valuable references for design and optimization.

**Fig. 15:**
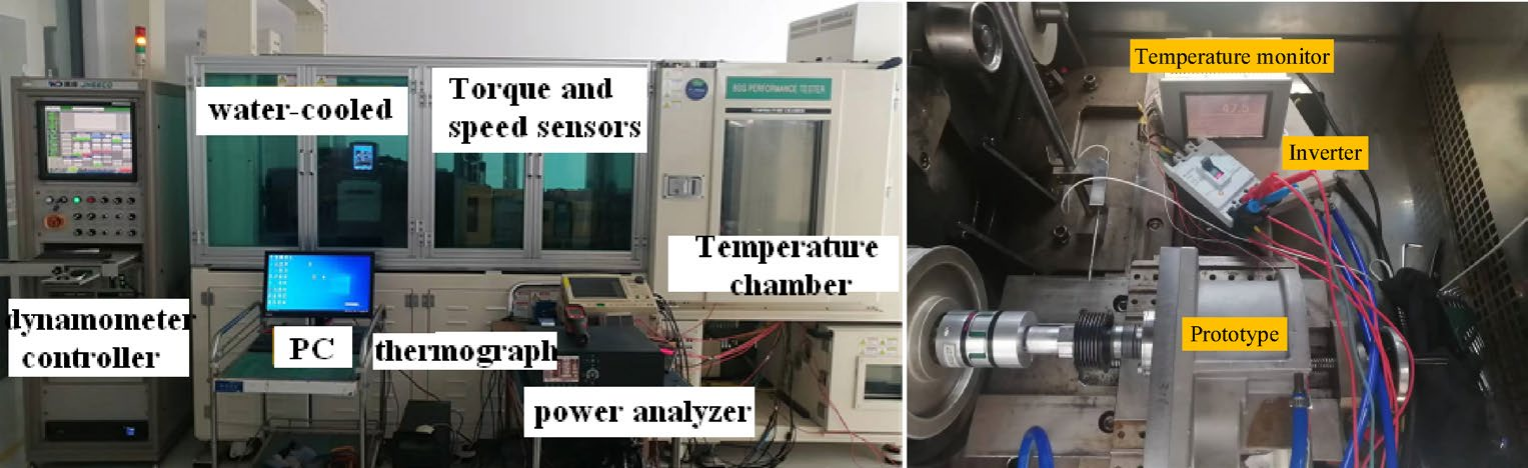
The test platform.

In addition, a testing platform was set up, as shown in Fig. 15. Figure 15 indicates that the test platform consists of the following components: the prototype, the inverter, the torque-speed tester, the dynamometer and its controller, the water cooling equipment and its control unit, and the measurement equipment (power analyzer, oscilloscope, temperature monitor, thermal imager, etc.). The inverter uses Kewo AD800N series high-performance vector inverter. The power analyzer is Fluke NORMA5000, and the oscilloscope is Yokogawa DL750. The platform was used to test the efficiency and power factor of the prototype under rated conditions.

In accordance with the IEEE Standard 1812 23, tests were conducted on the efficiency and power factor under rated load conditions, and the test results were compared with the simulation results gap between stator modules, as shown in Table 3.

**Table 3:** Prototype efficiency and power factor test Results.

Method	FEM	ICM	Test Results
Efficiency	75.3%	74.8%	69.5%
Power Factor	0.82	0.81	0.80

In Table 3, both the test results and the simulation results reflect the efficiency and power factor with gaps between stator modules. By comparing the test and simulation results in Table 3, it can be found that the results calculated using the ICM are more consistent with the experimental data than those obtained by the FEM. The ICM, after considering mechanical losses and permanent magnet losses, can accurately more reflect the actual operating conditions, and therefore, the calculated efficiency and power factor are closer to the experimental results. This finding further validates the accuracy and practicality of the ICM.

## Conclusion

In this study, the Finite Element Method (FEM) and the Iterative Calculation Method (ICM) were employed to investigate the impact of stator module gap variations on the performance of the CPMSM with segmented stators under constant current and constant output torque conditions. The findings indicate that the motor performance predicted by the ICM, accounting for mechanical and eddy current losses of permanent magnets, aligns more closely with experimental results compared to the FEM.

Specifically, under constant motor current, an increase in the stator module gap leads to a gradual decrease in core loss, can loss, torque, efficiency, and power factor, but torque ripple will also decrease. Conversely, under constant motor output torque, enlarging the stator module gap results in a gradual reduction in can loss, core loss, and power factor, while slightly enhancing the motor efficiency.

## Data Availability

The data used to support the findings of this study are included within the article.
